# Assessing Computational Methods of Cis-Regulatory Module Prediction

**DOI:** 10.1371/journal.pcbi.1001020

**Published:** 2010-12-02

**Authors:** Jing Su, Sarah A. Teichmann, Thomas A. Down

**Affiliations:** 1 MRC Laboratory of Molecular Biology, Cambridge, United Kingdom; 2 The Wellcome Trust/Cancer Research UK Gurdon Institute, University of Cambridge, Cambridge, United Kingdom; Memorial Sloan-Kettering Cancer Center, United States of America

## Abstract

Computational methods attempting to identify instances of cis-regulatory modules (CRMs) in the genome face a challenging problem of searching for potentially interacting transcription factor binding sites while knowledge of the specific interactions involved remains limited. Without a comprehensive comparison of their performance, the reliability and accuracy of these tools remains unclear. Faced with a large number of different tools that address this problem, we summarized and categorized them based on search strategy and input data requirements. Twelve representative methods were chosen and applied to predict CRMs from the *Drosophila* CRM database REDfly, and across the human ENCODE regions. Our results show that the optimal choice of method varies depending on species and composition of the sequences in question. When discriminating CRMs from non-coding regions, those methods considering evolutionary conservation have a stronger predictive power than methods designed to be run on a single genome. Different CRM representations and search strategies rely on different CRM properties, and different methods can complement one another. For example, some favour homotypical clusters of binding sites, while others perform best on short CRMs. Furthermore, most methods appear to be sensitive to the composition and structure of the genome to which they are applied. We analyze the principal features that distinguish the methods that performed well, identify weaknesses leading to poor performance, and provide a guide for users. We also propose key considerations for the development and evaluation of future CRM-prediction methods.

## Author Summary

Transcriptional regulation involves multiple transcription factors binding to DNA sequences. A limited repertoire of transcription factors performs this complex regulatory step through various spatial and temporal interactions between themselves and their binding sites. These transcription factor binding interactions are clustered as distinct modules: cis-regulatory modules (CRMs). Computational methods attempting to identify instances of CRMs in the genome face a challenging problem because a majority of these interactions between transcription factors remain unknown. To investigate the reliability and accuracy of these methods, we chose twelve representative methods and applied them to predict CRMs on both the fly and human genomes. Our results show that the optimal choice of method varies depending on species and composition of the sequences in question. Different CRM representations and search strategies rely on different CRM properties, and different methods can complement one another. We provide a guide for users and key considerations for developers. We also expect that, along with new technology generating new types of genomic data, future CRM prediction methods will be able to reveal transcription binding interactions in three-dimensional space.

## Introduction

### Cis-regulatory module definition

**Figure 1:**
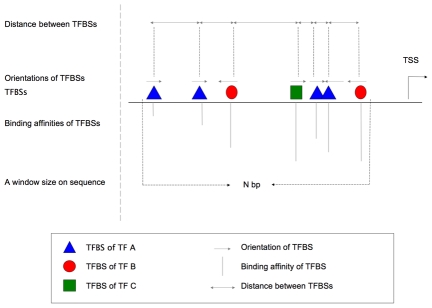
Schematic representation of cis-regulatory modules. A cis-regulatory module contains multiple binding sites of multiple transcription factors within a compact sequence interval. The binding affinity and the orientation of each binding site, the spacing and cooperation relationship between binding sites, and the relevant distance of cis-regulatory module to transcription start site of the gene it regulates may all be important properties of a given cis-regulatory module.

Cis-acting transcriptional regulation involves the sequence-specific binding of transcription factors (TFs) to DNA [1], [2]. In most cases, multiple transcription factors control transcription from a single transcription start site cooperatively. A limited repertoire of transcription factors performs this complex regulatory step through various spatial and temporal interactions between themselves and their binding sites. On a genome-wide scale, these transcription factor binding interactions are clustered as distinct modules rather than distributed evenly. These modules are called cis-regulatory modules. On DNA sequences, promoters, enhancers, silencers and others, are examples of these modules. The appropriate transcription factors compete and bind to these elements, and act as activators or repressors. Generally a CRM ranges from a few hundred basepairs long to a few thousand basepairs long; several transcription factors bind to it, and each of these transcription factors can have multiple binding sites (Figure 1).

Berman et al. [3] demonstrated the feasibility of identifying CRMs by sequence analysis. They scanned the *Drosophila* genome for clusters of potential binding sites for five gap gene transcription factors that are known to, together regulate the early *Drosophila* embryo. They found more than a third of the dense clusters of these binding sites correspond to be CRMs regulating early embryo gene expressions, characteristic of genes regulated by maternal and gap transcription factors. Similarly, exploiting the property that many CRMs contain clusters of similar transcription factor binding sites (TFBSs), Schroeder et al. [4] computationally predicted CRMs over the genomic regions of genes of interest with gap or mixed maternal-gap transcription factors, and identified both known and novel CRMs within the segmentation gene network.

Recent study has confirmed the importance of CRM functions, and revealed how subtle changes to the original arrangement of module elements can affect its function. Gompel et al. [5] found that modifications to cis-regulatory elements of a pigmentation gene *Yellow* can cause a wing pigmentation spot to appear on *Drosophila biarmipes* similar to that seen in *Drosophila melanogaster*, thus showing that mutations in CRMs can generate novelty between species. In a later study [6] they showed the creation and destruction of distinct regulatory elements of same gene can lead to a same morphological change. Williams et al. [7] investigated the genetic switch whereby the *Hox* protein *ABD-B* controls *bab* expression in a sexually dimorphic trait in *Drosophila*. They discovered the functional difference of this case lies not only in the creation and destruction of the binding sites, but also in their orientations and spacings. There is also evidence showing that disruption of cooperations within a specific CRM can lead to malformation and disease. One example is given by Kleinjan et al. [8]. The deletion of any distal regulatory elements of *PAX6* changes its expression level and causes congenital eye malformation, aniridia, and brain defects in human.

### Cis-regulatory module prediction methods

Methods attempting to identify CRMs in the genome face a challenging problem: a module is a mixture of signals – transcription factor binding sites and other sequence features – and these signals are spatially clustered within a specific genomic interval and are frequently, but not universally, conserved between related species [9]. Searching for a cis-regulatory module consists of searching for two properties: a set of signals, and the spatiotemporal relationships between this set of signals. In order to identify CRMs, one must first define and build a model.

Except for a small number of specific, well-characterized, interactions, the vast majority of spatiotemporal relationships between transcription factors remain unknown. This information deficit limits most CRM prediction methods to defining CRMs based on their general features: their spatial constraints (*i.e.* a close distance between binding sites within a CRM), their phylogenetic constraints (*i.e.* a CRM is a conserved block between species) [10]–[12], or both. Therefore, pre-compiled binding site profile libraries and multiple genome alignments are required by many CRM prediction methods.

**Figure 2:**
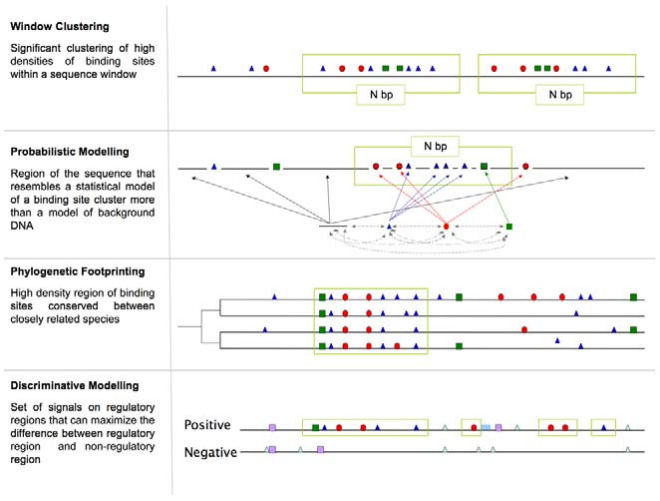
Classification of search strategies. Search strategies for the CRM prediction methods can be broadly subdivided into four families: window clustering, probabilistic modelling, phylogenetic footprinting, and discriminative modelling.

The search strategies for the existing methods can be roughly classified into four families. Window clustering involves significant clustering of high densities of binding sites within a sequence window. Probabilistic modelling consists of identifying sequences that resemble a statistical model of a binding site cluster more than a model of background DNA. Phylogenetic footprinting searches for high density regions of binding sites conserved between closely related species. Discriminative modelling seeks to identify set of signals on regulatory regions that can maximize the differences between regulatory regions and non-regulatory regions (Figure 2). Many methods are hybrids of two or more strategies.

### Assessment of methods

We wished to understand the performance of CRM prediction methods and, if possible, identify an optimal method. We also hoped to locate the key features that distinguish a good method and the reasons behind it. More specifically, we would like to answer these questions: (1) Which search strategy best predicts CRMs? (2) What types of CRMs are easy or difficult to predict? (3) What causes false positives and false negatives, and how they can be reduced in the future?

**Figure 3:**
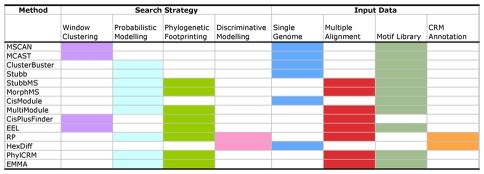
Properties of CRM prediction methods.

In order to examine all of these features of CRM prediction methods, we selected twelve representative methods from the above four search strategies families: MSCAN [13], MCAST [14], ClusterBuster [15], Stubb [16], StubbMS [17], MorphMS [18], CisModule [19], MultiModule [20], CisPlusFinder [21], phastCons score [22] (http://hgdownload.cse.ucsc.edu/goldenPath/dm2/phastCons9way/), Regulatory Potential [23] and EEL [24]. These twelve methods cover almost all the possible combinations of CRM representations, information resources used and search strategies available, as shown in the the summary table (Figure 3). Their operational principles are summarized (Table 1). Among these twleve methods, Regulatory Potential and EEL only have results available for the human genome. Therefore the other ten methods were applied to predict the CRMs in the *Drosophila* CRM database REDfly [25] to assess their general predictive power. Next, three optimal methods from the REDfly prediction result, together with Regulatory Potential and EEL, were applied to the human ENCODE regions, to assess the utility of these methods when dealing with different genomes of various compositions and structures.

**Table 1:** The operational principles of the methods based on cited publications.

Method	Operational Principle
MSCAN	Web server and source code: http://www.cisreg.ca/cgi-bin/mscan/MSCANMSCAN identifies binding site cluster with the significance of observed sites, while correcting for local compositional bias of sequence [13].
MCAST	Source code: http://metameme.sdsc.edu/doc/mcast.htmlMCAST searches for statistically significant cluster of non-overlapping matches to the query motifs [14].
ClusterBuster	Web server and source code: http://zlab.bu.edu/cluster-buster/ClusterBuster searches for regions that resemble a statistical model of a motif cluster more than a model of ‘background DNA’. The model of motif cluster is a uniform distribution randomly occurred motifs across the region, and the background model consists probabilities of independent, random nucleotides. It firstly performs one pass of the Forward algorithm to obtain the log likelihood score for each subsequence and keeps track of the subsequences with the maximal score. Secondly it performs the Backward algorithm for those tracked subsequences from their ends to their starts, to refine the optimal start point. At the end, it merges the tracked subsequences with a greedy algorithm [15].
Stubb	Web server and source code: http://stubb.rockefeller.edu/Stubb parses a CRM as a collection of binding sites interspersed with random bases, while considering correlations between binding sites. It assumes that a probabilistic process of hidden Markov model generates sequences. At each step, the process chooses either a motif at random or the background motif. The transition probabilities of the motifs and the background, and the correlated transition probability between pairs of motifs, are trained by the Expectation-Maximization algorithm to iteratively converge to a locally optimal [16].
StubbMS	Web server and source code: http://stubb.rockefeller.edu/The Stubb HMM framework is integrated with multiple species comparison by using sequence alignment as a first step. For two species, Lagan is used to find the best syntenic parse of ungapped conserved blocks. The binding site matches in the conserved blocks are evaluated using a HMM phylogenetic model. The unaligned sequences are computed as one single species and contribute independently to the final score of a homologous window [16].
MorphMS	Source code: http://veda.cs.uiuc.edu/Morphalign/supplement/MorphMS implements a pair-HMM statistical alignment method to generate alignments between two species. Therefore the uncertainty of alignment can be quantified probabilistically. The parameters except window length are estimated automatically from the input sequences. For each window, it then uses HMM model to generate orthologous CRMs. MorphMS produces two log likelihood ratio (LLR) scores for each position of input sequence: the LLR1 score compares the likelihood of a sequences is generated by mixture of motifs to the likelihood of this sequence is generated by the background model; LLR2 score shows the likelihood of the two orthologous sequences are generated independently [18].
CisModule	Source code: http://www.stat.ucla.edu/~zhou/CisModule/CisModule is a hierarchical mixture (HMx) model that describes CRMs in two levels: at the first level the sequences can be viewed as a mixture of CRMs interspersed by pure background sequences; at the second level, CRMs can be modelled as a mixture of motifs and within-module background. Bayesian inference is performed with Gipps sampling algorithm for the simultaneous detection of modules, TFBSs, and motif patterns, based on their joint posterior distribution [19].
MultiModule	Source code: http://www.stat.ucla.edu/~zhou/MultiModule/index.htmlMultiModule uses a hidden Markov model to model the co-localization of TFBSs within each species then couples the locations of TFBSs and modules through multiple alignments. Different evolutionary models are developed to capture the difference between the conservation of the TFBSs and the background. A Markov Chain Monte Carlo algorithm is developed to sample CRMs and their TFBSs simultaneously by their joint posterior distribution [20].
CisPlusFinder	Source code: http://jakob.genetik.uni-koeln.de/bioinformatik/people/nora/nora.htmlCisPlusFinder predicts CRMs by identifying high-density regions of perfect local ungapped sequences (PLUSs) based on multiple species conservation, with a second signal of locally overrepresented sequence motifs. The criterion of PLUSs to be selected is: the PLUSs contains at least one locally overrepresented core motif and there are additional PLUSs occur within the immediate neighbourhood [21].
EEL	Web server and source code: http://www.cs.helsinki.fi/u/kpalin/EEL/EEL locates the highest-energy elements by considering both conservation and biochemical and physical model of TF binding. The parameters contribute to the EEL score include both the binding affinities of the TFs to their respective binding sites and the distances between the adjacent binding sites. The difference on this distance between the two species alignments are also counted, so are the differences in the angle of the TFs [24].
RP	Source code: http://www.bx.psu.edu/projects/rp/RP identifies regulatory regions by statistically modelling frequencies of short alignment patterns in regulatory regions and background sequences. It describes two species alignments by five symbols: match involving A and T, match involving C and G, transition, transversion and gap. It classifies a set of k-mers of these symbols that are more overrepresented in regulatory regions than of neutral DNAs. The sequences are modelled by (k-1) Markov chain and the parameters are learnt from the experimentally confirmed regulatory regions and aligned ancestral interspersed repeats [23].
HexDiff	Source code: http://www.ics.uci.edu/~bobc/hexdiff.htmlHexDiff learns a set of hexamers that are more frequent occurred in known CRMs than non-CRMs, and applies them to predict CRMs in regulatory systems [28].
PhylCRM	Source code: http://the_brain.bwh.harvard.edu/PhylCRM/PhylCRM quantifies the clustering of the motifs identified by MONKEY in multiple alignments [29].
EMMA	Source code: http://veda.cs.uiuc.edu/emma/EMMA captures different evolutionary modes of TFBSs, and takes uncertainty of alignments and gains of losses of TFBSs into account. It uses a statistical alignment method and the substitutions are estimated by the HKY model [85]. For the TFBS evolution, it uses the population-genetic based Halpern-Bruno (HB) model [86]. It models the functional gains and losses of binding sites by switching the models that governs the evolutions of TFBSs and non-TFBSs, similar to [30], [87]–[89].

The family of window clustering methods, such as MSCAN, MCAST and CisPlusFinder, represent a CRM in a most naïve form as a statistically significant clustering of high affinity transcription factor binding sites. MSCAN and MCAST scan a motif library against a single genome. CisPlusFinder takes the perfect local ungapped sequences as potential transcription factor binding sites, then searches for a high density of multiple such short sequences that are conserved between closely related species.

The family of probabilistic modelling methods, ClusterBuster, Stubb, StubbMS, MorphMS, CisModule, and MultiModule, all implement a hidden Markov model (HMM) and they model a CRM sequence as being generated by a combination of a set of binding sites. The difference between them is ClusterBuster, Stubb and CisModule are based on a single genome while StubbMS, MorphMS and MultiModule are based on a pair or multiple orthologous genomes. Morever, the difference between StubbMS and MorphMS lies on their first step of aligning their input orthologous sequences: StubbMS uses Lagan [26] that produces a fixed alignment according to the sequence similarity. On the contrary, MorphMS aligns sequences by probabilistically summing over all possible alignments by their matches to the potential binding sites. CisModule and MultiModule are unique from the rest methods of this family by predicting both binding sites and CRMs in one step. CisModule encodes binding sites and a CRM into one hierarchical mixture model and follows Bayesian inference to predict both the location of CRM and the location of the binding sites within the CRM simultaneously. MultiModule follows the same model but improves on CisModule by incorporating information from comparative genomes.

Among the above two families of methods, the methods using multiple alignments: CisPlusFinder, StubbMS, MorphMS and MultiModule are also members of the phylogenetic footprinting family.

Among these ten methods, CisModule, MultiModule and CisPlusFinder are the three methods that do not rely on the prior information of a motif library. To further check how well the functional CRMs can be predicted without additional binding site knowledge, we applied a method based purely on sequence conservation – as represented by phastCons score [22] – as an independent calibration. PhastCons score is calculated by a phylogenetic hidden Markov model considering the evolutionary distance between species. It assigns each nucleotide position a score which represents the conservation degree of that position. We followed the approach used by King et al. [27] and took continuous windows with a mean phastCons score over an optimized phastCons score threshold as a potential CRM (see Materials and Methods).

We also identified a few interesting methods which we were unable to include in this assessment due to incompatibility with the experimental design of this study or unavailability of required data. For example HexDiff [28], a method in the discriminative modelling family, learns a set of over-represented hexamers in known CRM sequences, and discriminates CRM sequences from non-CRM sequences by searching for the highest frequency hexamers. Such a method requires correctly annotated positive and negative datasets of known CRMs to assess its performance. Regulatory Potential [23] is another type of discriminative method, which learns the abundant hexamers and the first order dependency relationships between columns of aligned position from known regulatory regions. Similar to MorphMS are EEL [24], PhylCRM [29] and EMMA [30], which aim to better use multiple genome information by implementing binding site-based alignment methods. EEL considers the potential secondary structure of a DNA-protein complex by weighting the difference in the distance between adjacent binding sites between the two aligned species. PhylCRM uses MONKEY [11] directly to predict true functional binding sites in its first step. MONKEY uses multiple alignments and models the binding sites of each transcription factor with a specific evolutionary model. Thus, the binding sites predicted by MONKEY are enriched for true conserved functional sites among those gained, lost and turned over. EMMA takes a similar approach as MorphMS but incorporates binding site gains and losses. However, this makes its computational cost increase exponentially with the number of transcription factors considered, and limits EMMA to more focused problems, rather than genome-wide studies.

### Other methods and previous assessments

There are a number of studies that search for tissue specific or stage specific CRMs based on a set of co-regulated genes. Some studies also include information from microarray expression data, such as LRA [31], ClusterScan [32], Composite Module Analyst [33], and ModuleMiner [34]. Other methods scan only for regions where a small set of user defined transcription factors bind but do not predict novel CRMs, such as STORM & MODSTORM [35], ModuleScanner [36], Target Explorer [37], and CisModScan [38]. These types of methods are not included in this assessment because we focus on genome wide novel CRM prediction methods.

Several previous publications have reviewed different aspects of some of these methods. Gotea et al. [39] studied the problem on a small scale up to 10kb upstream of sets of co-expressed genes; Aerts et al. [40] performed a genome-scale target genes prediction for individual transcription factors; King et al. [27] compared methods using comparative genomics in different ways; Wang et al. [41] experimentally validated predictions based on the hypothesis that the combination of high Regulatory Potential and existence of a conserved known binding motif is a good predictor for functional CRMs; Halfon et al. [42], Chan and Kibler [28] and Pierstorff et al. [21], each compared the performance of several CRM prediction methods. However, their results are based on several small sets of data and the number of methods compared is limited.

## Results

Our scenario for using CRM prediction tools involves taking either a complete, unannotated genome, or a large genomic interval, and running the tools to identify candidate regulatory regions. Evaluating methods in this scenario is difficult because there are few large genomic regions where we are certain that all regulatory elements have been discovered. Thus it is hard to accurately estimate the false positive rate. To compose a test dataset for this experiment, we prepared one true positive dataset of known CRMs from REDfly - a curated collection of experimentally validated *Drosophila* transcriptional cis-regulatory modules and transcription factor binding sites, and two true negative datasets of known non-regulatory sequences: introns and exons from the *Drosophila melanogaster* genome (see Materials and Methods). It has been reported that some cis-regulatory elments do exist in long introns, especially first introns [43]–[45]. To further eliminate such contamination from the negative intron dataset, we assembled only short introns which are smaller or equal to 81 bp [46] into the negative dataset. To reflect the performance of these methods when facing the entire genome, we also assembled two additional datasets from medium length introns and intergenic regions (see Materials and Methods). The ten selected methods were applied to predict CRMs against intron, exon and intergenic sequences. The intrinsically different compositions and characteristics of these sequences affect the prediction of these methods. `

Each method was applied with – as near as practical – its default parameter settings. Most methods have a default window size of either 200 bp or 500 bp. To avoid any bias toward a specific window size setting, and to understand which size is a more general representation of real CRMs length, each method was repeated with both window size settings.

For those methods requiring double or multiple alignments, the alignments of *Drosophila melanogaster* and *Drosophila pseudoobscura* were retrieved from the MAVID [47] multiple alignments server (http://www.biostat.wisc.edu/~cdewey/fly_CAF1/), and the alignments of *Homo sapiens* and *Mus musculus* are retrieved from the UCSC genome browser (http://hgdownload.cse.ucsc.edu/goldenPath/hg18/encode/MSA/DEC-2007/sequences/). For those methods requiring a motif library, we used 68 motifs of *Drosophila melanogaster* from the Transfac transcription factor database version 10.4 [48]. For the predictions on ENCODE data, we used the set of 107 human motifs compiled and used by the EEL developers in their work [24] (http://www.cs.helsinki.fi/u/kpalin/EEL/data/).

### The REDfly database CRMs: Ranking of methods

The results of the ten selected methods on REDfly are plotted as a receiver operating characteristic (ROC) curve, where sensitivity is plotted as a function of specificity at different cut-off thresholds. Sensitivity is proportional to the true positive rate indicating how many true CRMs are found from all the annotated CRMs, (Sensitivity = TP/P = TP/(TP+FN)). Specificity depends on the true negative rate indicating how many true introns, exons or intergenic regions are found from the negative dataset (Specificity = TN/N = TN/(TN+FP)). The ten methods applied are in ten different colours. Each method has two ROC curves, one is for window size 200 bp, and another one is for window size 500 bp.

**Figure 4:**
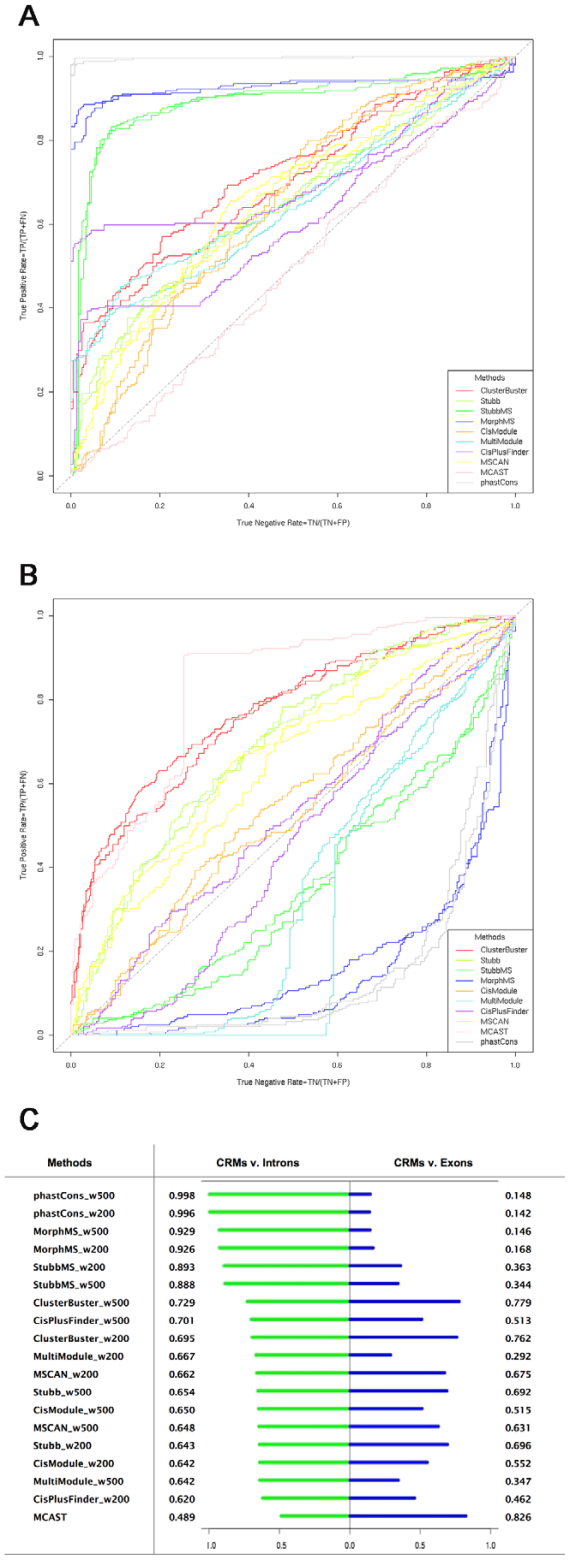
Ranking of methods (short introns and exons). A. Predictions of CRMs against short introns. There are two ROC curves for each method, one for 500 bp and one for 200 bp window size. B. Predictions of CRMs against exons. There are two ROC curves for each method, one for 500 bp and one for 200 bp window size. C. Ranking of methods by Area Under Curve scores.

The ROCs of the methods' ability to distinguish CRMs from short introns are plotted (Figure 4A). All methods show a positive predictive power, except MCAST whose prediction power is close to random. The results show two clear clusters: the methods based on a single genome, and the methods based on multiple genomes. Among the single-genome methods, the best performing one is ClusterBuster. Among the multiple-genomes methods, the best performing one is MorphMS. Among all the ten methods, StubbMS and MorphMS outperform the other methods clearly. The fact that MorphMS performs better than StubbMS suggests that a probabilistic alignment strategy based on binding sites does capture the functional element information better than the conventional alignment strategy based on nucleotides, as stated in Sinha and He [18].

CisPlusFinder and MultiModule are based on multiple genome alignments and do not show any dramatic improvement over the single-genome methods. CisPlusFinder performs well while its CRM score threshold is high, but it deteriorates as the threshold is reduced. This might be due to the specific type of CRM targets of CisPlusFinder: CisPlusFinder defines a CRM as a cluster of so called perfect local ungapped sequences – multiple copies of over-represented binding sites in a single sequence. Each set of perfect local ungapped sequences is a homotypical clustering of binding sites of one transcription factor, and a cluster of these sequences refers to the CRMs containing multiple homotypical clusterings of binding sites. Thus the CRMs containing only binding sites of a single transcription factor, or a heterotypical cluster of several single binding sites, will be missed. Another factor that may affect their performance is that these two methods do not use a motif library, unlike StubbMS and MorphMS, as predicting both the transcription factor binding site and the CRM simultaneously is a more challenging task. Unexpectedly, the simple peak phastCons score window method outperforms all the more complex methods. When evolutionary conservation is used as an independent feature to distinguish the true CRMs from the intronic sequences, its performance is nearly perfect.

The ROCs of the methods distinguishing CRMs from exons are plotted (Figure 4B). This result shows a dramatic reversal of the curves of those methods based on multiple alignments, indicating that these methods are driven heavily by the conservation feature of the given sequences and do not have the ability to distinguish conserved regulatory elements from conserved protein-coding sequences. This also indicates that there are many false positive hits of transcription factor binding sites on exon regions as well, as a motif library of known transcription factor binding sites is not able to compensate for the high level of sequence conservation. The more a method relies on the conservation factor when predicting CRMs, the worse it performs at distinguishing CRMs from exons. That is why the peak phastCons score window method performs the worst in this case. The only exception is CisPlusFinder, which does not fall completely into the bottom right space. CisPlusFinder requires a candidate CRM sequence not only to be conserved, but also has the inter-relationships between the adjacent perfect local ungapped sequences. Only a cluster of the local ungapped sequences can be the CRM candidate. This condition reduces the likelihood of conserved exon sequences being recognized falsely as functional regulatory sequences. However, it still loses its prediction power as the score threshold goes down. On the contrary, the methods based on a single genome stay at a similar level to their results on distinguishing the CRMs from the introns, and the optimal one is still ClusterBuster.

To summarize, for the ROC curves above, an Area Under ROC Curve (AUC) score is calculated as a representation of the prediction power of a method. Then the methods are ranked by their AUC scores according to their results of distinguishing the CRMs from the short introns (Figure 4C). The top three performing methods are all multiple alignments based: phastCons, MorphMS and StubbMS. However, they all show a weak predictive power against exons. ClusterBuster ranks fourth for its predictions against short introns. Compared to the first three methods, its performance is similar against both short introns and exons. Given an unannotated genome, such a method will provide more reliable predictions.

For most of the selected methods under this experimental setting, their predictions are not very sensitive to the window size 200 bp or 500 bp settings. The probabilistic modelling methods, especially the ones using multiple genomes, such as StubbMS, are less sensitive than the window clustering methods, such as CisPlusFinder. CisPlusFinder performs better when its window size is set to be 500 bp instead of 200 bp: a longer region is more prone to have multiple homotypical clusterings as CisPlusFinder targets for. The slightly preferred window size for majority of methods is 500 bp, which is similar to the average length 635 bp of predicted human and mouse CRMs of the database PReMod [49], [50], and the average length 760 bp of fly CRMs of the database REDfly [51].

**Figure 5:**
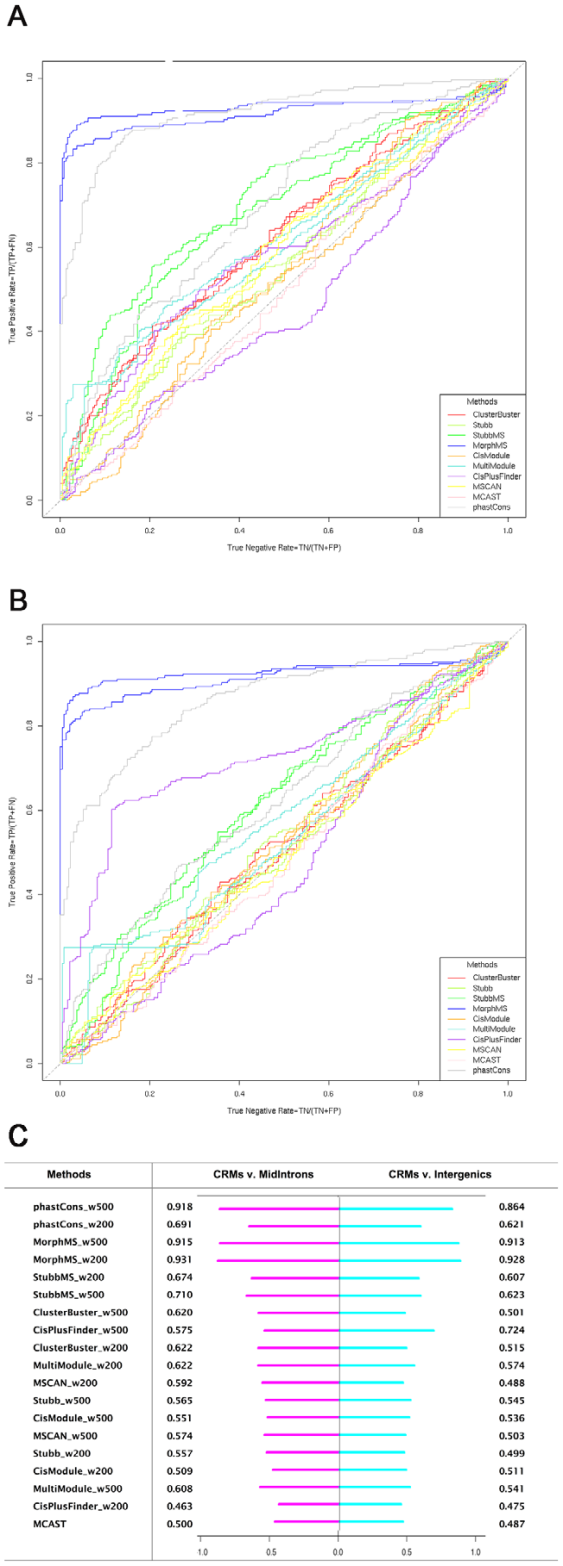
Ranking of methods (medium length introns and intergenic regions). A. Predictions of CRMs against medium length introns. There are two ROC curves for each method, one for 500 bp and one for 200 bp window size. B. Predictions of CRMs against intergenic regions. There are two ROC curves for each method, one for 500 bp and one for 200 bp window size. C. The Area Under Curve scores of the assessed methods.

We also obtained the prediction results of these ten methods on a medium length intron dataset (Figure 5A) and an intergenic dataset (Figure 5B)(Figure 5C. the AUC scores of the assessed methods). All methods except MorphMS and MCAST, show a clear performance deterioration compared to the short intron dataset. This is not surprising considering that the medium length introns and the intergenic regions are more likely to contain actual transcription factor binding sites than the short introns, and the intergenic regions are the most contaminated among these three regions [52], [53]. This is illustrated clearly by the performance changes of the methods relying on clusters of binding sites only, such as ClusterBuster, Stubb and MSCAN. The phastCons score window method performed much worse on these two datasets than on the short intron dataset. The gap between the predictions of the window size 200 bp setting and the prediction of the window size 500 bp setting is significantly larger than their difference on the short intron and the exon datasets. The result of the 500 bp window size is superior to 200 bp. It is known that introns can mediate gene expression in various ways [54]. The intron length is connected to alternative splicing events (http://www.sdbonline.org/fly/aimain/6rna-ooc.htm) and functional introns tend to be the larger ones [55]. The conserved intergenic regions are also known to play regulatory roles [56]. Therefore it is very likely that there are conserved functional regions existing in the medium length introns and intergenic regions, and some of them can span around 200 bp. CRMs can be distinguished from these functional regions by a larger window size setting of 500 bp. Apart from above differences, these results agree with those obtained from the short intron dataset in terms of ranking among the methods and similar performance between the two window size settings for each method.

### Correlations of methods

**Figure 6:**
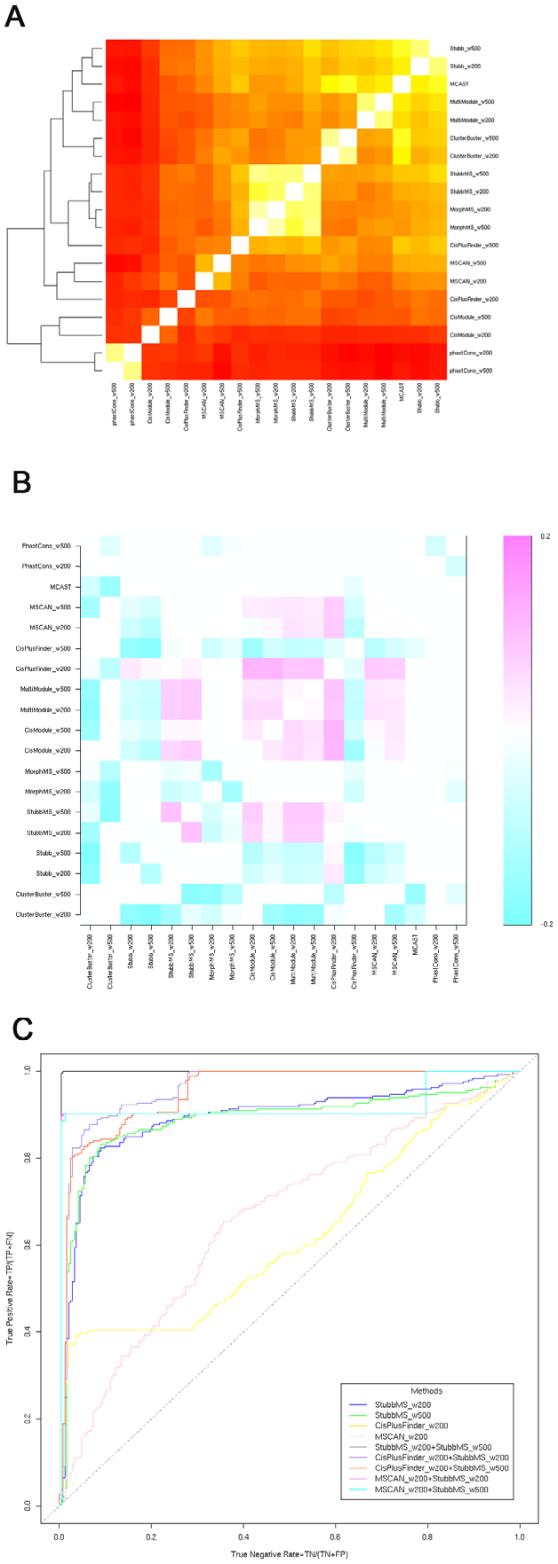
Correlations and complementarity of methods. A. Correlation coefficients of predictions on CRMs. B. Performance of pairs of methods. C. Improvement made by combining pairs of methods: StubbMS_w200 and StubbMS_w500, CisPlusFinder_w200 and MSCAN_w200 to StubbMS_w200 and StubbMS_w500.

Based on the prediction score of REDfly CRMs given by each method, we normalized the scores of each method to the same scale between 0 to 1, by dividing each score by the maximum possible of that method. We then calculated the correlation coefficients between all pairs of methods (Figure 6A). For each method, the results with 200 and 500 bp window sizes correlate closely. Particularly for MorphMS, a very high correlation exists between the two predictions of these window sizes. This further confirms the previous results that these methods are not very sensitive to the window size parameter setting under this experimental design. One exception is CisPlusFinder, which shows a stronger prediction power with 500 bp window size compared to 200 bp. The other exception is CisModule, where the 200 and 500 bp window size results form two separate clusters. This might be explained by the fact that CisModule follows a non-deterministic algorithm and each run returns a slightly different result.

The high correlation coefficients show the agreement between these representative methods. Those methods with the same underlying CRM representations and which require the same prior information are clustered together as expected (*e.g.* MorphMS and StubbMS, CisPlusFinder and MSCAN, and ClusterBuster and Stubb). Unexpectedly, CisPlusFinder performs more similar to the multiple alignments probabilistic modelling methods StubbMS and MorphMS when its window size is set to be 500 bp. These three methods from two different families all have strong predictive power with significant agreement, despite their different underlying mechanisms. Another exception is MultiModule, which is clustered into the single genome probabilistic modelling family together with ClusterBuster and Stubb. MultiModule itself is a generative probabilistic model, similar to a hidden Markov model. However, the information from the double alignment does not improve the performance of MultiModule over the methods using a single genome only.

### Complementarity of methods

Pairwise complementarity of methods is checked by summing the normalized scores given by each pair of methods for both their predictions on the CRMs and their predictions on the short intron negative dataset. The increase or decrease of the AUC scores of the new pairs over the maximum of the individual methods is shown (Figure 6B).

Most methods deteriorate when the predictions of two different window size settings are summed together. This is clearly the case for MorphMS and the peak phastCons score window method. At the same time several other methods show an opposite effect, such as StubbMS for which the summed result brings its prediction power from AUC score 0.893 and 0.888 to 0.996 (Figure 6C). The new result is equivalent to the prediction power of phastCons score and is nearly perfect.

Amongst these methods, the window clustering family methods CisPlusFinder and MSCAN, especially with the window size 200 bp setting, are highly complementary to probabilistic modelling family methods StubbMS, CisModule and MultiModule. The performances of these pairs of methods are better than any individual method. One possible reason might be the different approaches of these methods to defining the candidate binding site profiles. CisPlusFinder is not constrained to the prior knowledge of binding site profiles and therefore has the potential to search for unknown transcription factor binding sites. Another reason might be that they focus on different length CRMs: the probabilistic modelling family methods tend to find short CRMs, while CisPlusFinder tend to find long CRMs. For example, the first quartile and the third quartile of the lengths of the predicted clusters by ClusterBuster with window size 200 bp setting are 149 bp and 790 bp accordingly; in the results of CisPlusFinder with window size 200 bp setting, there are only two predicted CRMs shorter than 200 bp, and the first quartile and the third quartile of the lengths of the predicted clusters are 677–1643 bp accordingly.

### Sequences features affecting predictions

**Figure 7:**
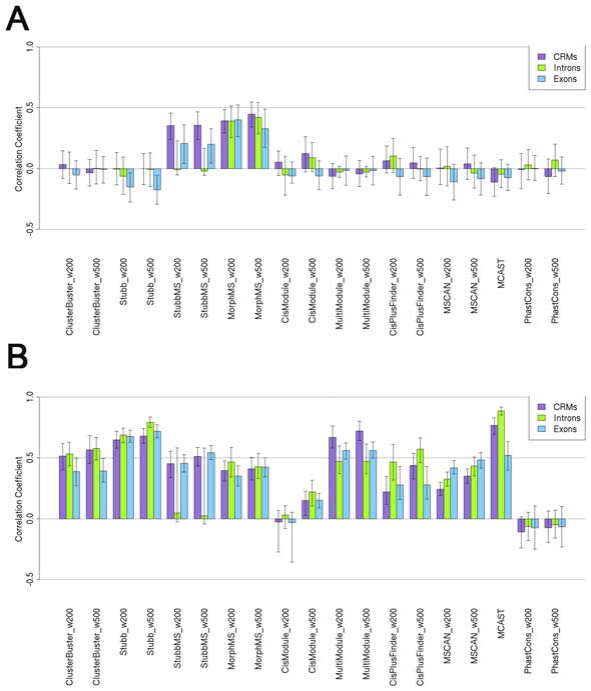
Correlation coefficients between predictions and sequence features. A. Correlation coefficients between predictions and sequence conservations, with 95% bootstrap confidence interval. B. Correlation coefficients between predictions and sequence lengths, with 95% bootstrap confidence interval.

To understand what properties of a CRM make it distinctive, and what features of a negative sequence cause false positive predictions, we checked the correlation coefficients between sequence features of the CRMs, the short introns and the exons, and the scores given by each method. The sequence features considered include its average conservation degree measured by phastCons score and its length (Figure 7).

The predictions of StubbMS and MorphMS are heavily affected by the average conservation degree of a sequence. This confirms that the high average sequence conservation is the key feature these two methods rely on, and it contributes both the true positives and false positives. The peak phastCons score window method, searching for continuous windows over a threshold, does not rely on this feature of CRMs for its prediction. The phastCons score window method predicts CRMs better than MorphMS and StubbMS, showing that searching for peak conservation regions on a sequence can capture more regulatory elements than counting the average sequence conservation.

For the correlations between the prediction and the sequence length, which is equivalent to the CRM length in this experimental design, nine out of ten methods show a correlation to a certain degree. Especially MultiModule, Stubb and ClusterBuster, the members of the probabilistic modelling family, have correlation coefficients over 0.5. Among all, MCAST is the method driven by the sequence length most. Basically, a long sequence brings a high scored prediction. This bias causes false positives of all the methods except the peak phastCons score window method, which does not rely on this feature of CRMs for its prediction.

We sorted the CRMs by the summed scores of all ten methods and excluded the CRMs having a 0 score by any method, and then checked the properties of the CRMs commonly found by the ten selected methods. These CRMs tend to be long sequences, but not always very conserved. The correlation coefficient between the predictions and the sequence lengths is high, while the same figure for the average sequence conservation is low. For the same reason, the false positive predictions from the short intron and the exon datasets also tend to be long sequences, and the correlations between the prediction and the sequence length are high. The peak phastCons score window method is the one least biased from these sequence features.

In summary, for most methods, long length and general conservation of a short intron or exon sequence contribute the most to both true and false positives. A continuous peak conserved window is a more distinctive and unique feature of a CRM, and can be used to identify the real CRMs as the success shown by the peak phastCons score window method.

### CRM properties affecting predictions

Among all the CRM sequences, 19 sequences are annotated with known transcription factors, and their transcription factor binding sites are experimentally validated and annotated by the *Drosophila* DNase I footprint database FlyReg [57]. This provides us a chance to further check how CRM properties affect the prediction of each method, based on the known information so far.

**Figure 8:**
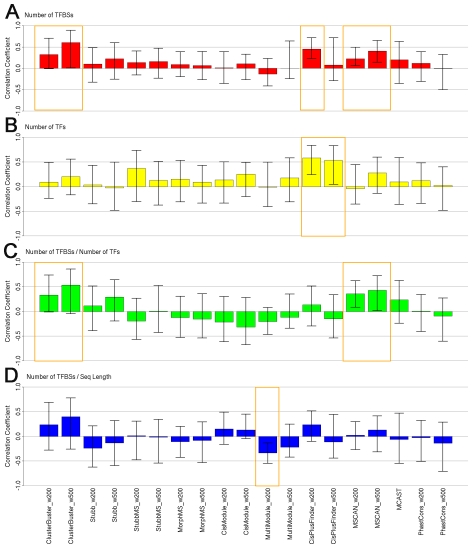
Correlation coefficients between predictions and CRM properties. A. Correlation between predictions and the total number of TFBSs, with 95% bootstrap confidence interval. B. Correlation between predictions and the total number of TFs, with 95% bootstrap confidence interval. C. Correlation between predictions and the number of TFBSs/number of TFs, with 95% bootstrap confidence interval. D. Correlation between predictions and the number of TFBSs/sequence length, with 95% bootstrap confidence interval.

We checked for how these methods are prone to the abundance of transcription factor binding sites, the number of transcription factors, and the composition of homotypical clustering, by calculating the correlation between the CRM properties and the prediction scores on the 19 annotated sequences (Figure 8). Different CRM representations and search strategies rely on different CRM properties. The predictions of the ClusterBuster, CisPlusFinder with window size 200 bp setting and MSCAN are significantly correlated with the total number of transcription factor binding sites of a CRM. CisPlusFinder also shows a strong correlation with the number of transcription factors a CRM contains. Indeed, it predicts the CRMs with multiple transcription factors only. The CRMs containing large homotypical clustering of multiple transcription factor binding sites are more likely to be found by ClusterBuster and MSCAN. For MultiModule, the density of transcription factor binding site on a sequence is critical for its prediction.

Some types of CRMs are easier to be predicted and some types of CRMs do not have very distinctive features (Table S1). The CRMs with multiple transcription factor binding sites of known transcription factors are easier to be predicted, such as CRM *Ubx_basal_promoter* containing 20 transcription factor binding sites of seven known transcription factors including *Ubx* and *zen*. Most methods score it high, especially ClusterBuster and CisPlusFinder with window size 200 bp setting. On the contrary, short CRMs with a few transcription factor binding sites are easily missed by most prediction methods. For example, for the 227 bp long *ninaE_distal_enhancer* with only two *gl* binding sites, ClusterBuster with window size 200 bp setting scores it very low because of there is not a profile of the *gl* transcription factor binding site supplied. CisPlusFinder scores it 0 for another reason: this CRM is composed of only one homotypical clustering. For the short CRMs with few transcription factor binding sites, the peak window phastCons score method will not miss it. For this particular CRM, phastCons with window size 200 bp setting scores it high as 0.991.

The peak phastCons score method does not always pick up the real CRMs. There are cases where the probabilistic modelling methods predict correctly while the peak phastCons score method does not. For example, CRM *Dpp_BS1.0* contains five transcription factor binding sites of transcription factor *en* within a 246 bp distance. The peak phastCons score window method scores it relatively low, while probabilistic modelling methods such MorphMS score this sequence high. The reason leading to this phenomenon could be the binding sites on this sequence are conserved but the sequence between them are not. Therefore there is not a continuous peak conserved window as the peak phastCons score method requires. MorphMS is able to detect such shifted conservation by aligning sequence by the location of transcription factor binding sites.

Unexpectedly, there are also cases where CisPlusFinder misses out genuine CRMs with multiple homotypical clusterings: such as *Ance_race_533*, a 533 bp long CRM annotated with nine transcription factor binding sites of three transcription factors including *Mad* and *zen*. Both CisPlusFinder with 200 bp window setting and with 500 bp window setting score this sequence as 0. The perfect local ungapped sequences defined by CisPlusFinder cannot always represent real binding sites accurately.

### Evaluation on human ENCODE regions

The above success of using pure conservation scores to predict CRMs suggests that searching for appropriately sized conserved blocks is sufficient to distinguish true CRMs from the REDfly database from short introns and exons. This may not be surprising considering the *Drosophila* genome is relatively small and compact, and its regulatory regions are closely packed together [58]. REDfly is principally composed of developmental enhancers and these elements are known to be generally very conserved [59], [60]. However, the dramatic contrast of the performance of these multiple alignment based methods depending on whether introns or exons are used as representative negative sequences leads us to question whether the level of conservation seen in the CRMs collected by REDfly is representative of typical CRMs. To further investigate this possibility and to check if these methods are sensitive to the composition and structure of the genome, we applied the optimal methods among the prediction on REDfly: ClusterBuster, MorphMS and the peak phastCons score (http://hgdownload.cse.ucsc.edu/goldenPath/hg18/phastCons17way/) window method, plus the peak Regulatory Potential score (http://hgdownload.cse.ucsc.edu/goldenPath/hg18/regPotential7X/) window method (see Materials and Methods) and the prediction results of EEL (http://www.cs.helsinki.fi/u/kpalin/EEL/), to human ENCODE regions. The human genome is more diverse on its conservation degree of regulatory elements. Specifically, 30 out of 44 ENCODE regions were picked by the ENCODE consortium according to their non-exonic conservation levels (1.1–6.2%, 6.3–10.2%, 10.7–18.6%.) and gene densities (0–1.7%, 2.0–3.6%, 4.4%–10.6%) [61] (http://genome.ucsc.edu/ENCODE/regions.html). We used these 30 ENCODE regions to make sure that the sequences are diverse in their converstaion degrees and thus eliminate the possibility of any bias caused by conservation.

**Figure 9:**
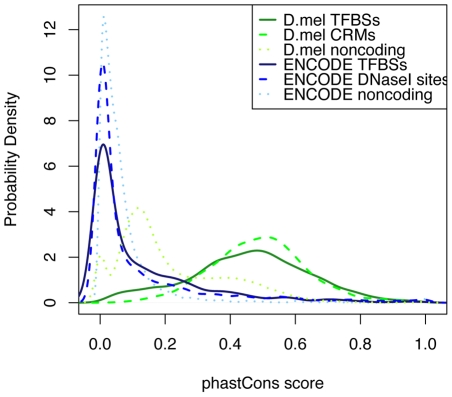
Comparison between the conservation degrees of transcription factor binding sites, CRMs and noncoding regions of Drosophila genome and human ENCODE regions. The probability density shows that, for *Drosophila*, the REDfly CRMs are more conserved than the transcription factor binding sites. For human ENCODE regions, the transcription factor binding sites are more conserved than the DNaseI hypersensitive sites.

Firstly we compared the conservation degree of transcription factor binding sites, CRMs, and noncoding regions of both *Drosophila* genome and human ENCODE regions (Figure 9). For human ENCODE regions, we used ENCODE regulome DNase I hypersensitive sites of human lymphoblastoid cells GM06990 [62] (http://hgdownload.cse.ucsc.edu/goldenPath/hg18/encode/database/encodeRegulomeDnaseGM06990Sites.txt.gz) as the potential CRMs which mark the chromatin regions having high accessibility to transcription factors. We expect the CRMs are less conserved than the transcription factor binding sites because CRMs contain less constrained sequences between transcription factor binding sites. The probability density shows that, for *Drosophila*, the REDfly CRMs are more conserved than the transcription factor binding sites. For human ENCODE regions, the transcription factor binding sites are more conserved than the DNaseI hypersensitive sites. This confirms that the REDfly CRMs are more conserved than expected. Comparing between the two genomes, the entire *Drosophila* genome and their regulatory regions are more conserved than their equivalents on the human ENCODE regions.

**Figure 10:**
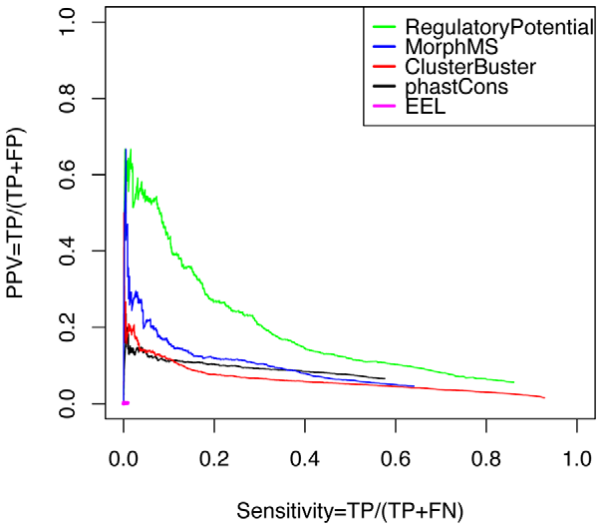
Predictions on ENCODE regions. The performance of methods ranks them from top to bottom in this order: Regulatory Potential, MorphMS, ClusterBuster, phastCons score, and EEL.

Next, we applied the five selected methods on the ENCODE regions, and their performances were evaluated by their overlaps with the DNaseI hypersensitive sites. If a prediction overlapped – even partially – with any DNaseI hypersensitive site, it was counted as a true positive. A prediction not overlapping with any DNaseI hypersensitive site was counted as a false positive. The missed DNaseI hypersensitive sites were counted as false negatives. Because these methods need to scan large ENCODE regions therefore it is not sensible to define a fixed-sized true negatives. For this reason, instead of specificity, positive prediction value was calculated to show the methods performance. The results of these methods were plotted in a pseudo ROC plot, where sensitivity is plotted against positive prediction value (PPV): sensitivity = TP/(TP+FN), indicating how many true CRMs are found among all the DNaseI hypersensitive sites, and PPV = TP/(TP+FP), indicating the percentage of true CRMs among all the predictions (Figure 10). Among all the methods, the peak Regulatory Potential score window method significantly outperforms the rest of the methods. This suggests that the information learnt from the known regulatory regions is very helpful indeed. Unexpectedly, EEL does not pick up any positive signals and is at the bottom of the chart. This might be due to the public available prediction results of EEL were produced with a high cut-off threshold, while the other methods' cut-off thresholds were deliberately set to be their lowest in this assessment to allow the maximum number of predictions. Overall, their performance ranks them from top to bottom in this order: the peak Regulatory Potential score window method, MorphMS, ClusterBuster, the peak phastCons score window method, and EEL. This result shows a different prediction power of some methods from their previous prediction performances of the REDfly CRMs: the peak conservation phastCons score approach does not outperform probabilistic modelling methods in this case.

For the window size setting (Figure S1), both ClusterBuster and MorphMS predictions with 500 bp window setting discovered slightly more CRMs than their predictions with 200 bp window setting, with a price paid by vastly increased computational time for MorphMS. We also increased the window size of the peak phastCons score window method and the peak Regulatory Potential score window method from 100 bp to 200 bp, 500 bp, 1000 bp and 1500 bp. The increase of the window size universally increased the performance of these two methods. Perhaps understandably, the optimal window size setting of these methods tuned for the human genome tend to be larger than the ones for the *Drosophila* genome.

Upon summarizing the above results, it is clear that the application of the prediction methods on the *Drosophila* genome and the human genome need to be treated differently. Not only are the composition [63] and the structure [64] of the *Drosophila* and human genomes different, but the evolutionary distance between the given alignment: *Drosophila melanogaster* and *Drosophila pseudoobscura*, human and mouse, are different too. The nucleotide conservation levels between the *Drosophila melanogaster* genome and the *Drosophila pseudoobscura* genome are ∼70% for coding sequences, ∼40% for introns [65]. The corresponding figures between the human and the mouse genomes are: ∼85% for coding regions, ∼35% for introns [66]. These might all contribute to the different performances of the prediction methods.

## Discussion

### Pros and cons of the existing methods

The two most frequently used types of genome information resources: conservation and transcription factor binding site profiles, and the four families of search strategies, are applied in numerous ways. Any subtle change in the combination or the order may yield different results. Therefore the existing methods can show a great variety of results given the same data. Although there is not a universal optimal method suitable for all situations, several key strategies applied in the existing methods do show their values on improving predictions.

The advantage of MorphMS over StubbMS for predicting REDfly CRMs supports the view that aligning multiple genomes by locations of conserved transcription factor binding sites can perform better than conventional alignment according to the nucleotides. CisPlusFinder can complement several methods. This brings our attention to neighbourhood relationships between homotypical clusters of sites for multiple factors. The success of the peak Regulatory Potential score window method shows the importance of the information learnt from the known regulatory elements, particularly, the novel strategy of considering the alignment pattern: the first order dependent relationship between the conserved columns within a transcription factor binding site.

**Figure 11:**
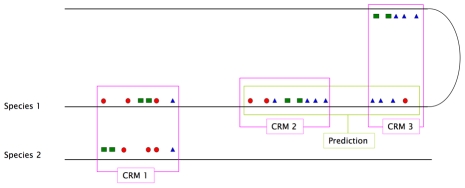
The majority existing methods target regions rich of cis-regulatory elements. Existing methods predict CRMs based on their distance and conservation features. This fact limits their targets are regions rich of closely located and highly conserved *cis-regulatory elements* (green region) instead of real functional modular CRMs (pink regions). Consequently, they will miss out: those CRMs composed of elements not conserved in a same order, e.g. CRM 1; those CRMs not conserved, e.g. CRM 2; or those CRMs composed of further apart elements, e.g. CRM 3. At the same time, uncompleted regions within a CRM, or overlapped regions shared by more than one CRMs, could be predicted as a false positive, e.g. a false positive prediction composed of CRM 2 and part of CRM 3.

However, there remain some clear problems with CRM prediction. Firstly is the fundamental problem of modelling functional CRMs: the majority of existing CRM prediction methods target regions rich in clustered and conserved transcription factor binding sites, and while this does work to a degree, it remains a relatively poor proxy for identifying functional regulatory elements (Figure 11). The fact is that the distance and conservation features of a sequence are not sufficient to accurately deduce its function. In addition, not all CRMs are tightly packed or highly conserved. At the same time, a fragment of a CRM, or overlapping regions shared by more than one CRMs, could be predicted as one complete CRM. Clearly, the current CRM prediction methods are only a first step towards accurately predicting true CRMs.

Secondly, the general CRM properties are not universally applicable. There are also exceptional cases where some real regulatory functional sites are not more conserved than the background sequences [67]. At the same time, not all the clustered conserved elements are cis-regulatory elements - they can be conserved non-functional noncoding regions [68], or other conserved signals which have other functions other than being an enhancer, e.g. microRNA. In addition, some transcription factors, such as *E2F1*, do not require a canonical binding site [69]; while for some other cases, for a same consensus, several transcription factors can compete each other on binding on it. Further more, the interactions between DNA and transcription factor, and the interactions between factors and factors form 3D complexes; this makes identifying the members indirectly involved even more difficult. Obviously, the information of binding affinity, the distance and the conservation, are far from being enough to identify a functional module.

Thirdly, the CRM prediction methods development and evaluation lacks genome-scale standardization and benchmarking. Most development and comparison on the CRM prediction methods were based on either a small set of genes or REDfly. King et al. [27] used *HBB* gene complex; Wang et al. [41] used the mammalian genes expressed in read blood cells; or Aerts et al. [40] and Sinha and He [18] used REDfly, which is the only experimentally confirmed genome wide CRM database available. A small set of co-expressed genes tends to have a limited number of similar CRMs made of a few transcription factors, and we showed that the CRMs in the REDfly database are very conserved and therefore might not able to represent the general CRMs on other genomes. A method tuned on the maximum performance on these sequences can be biased toward the extreme properties of the data itself and therefore is not suitable to be universally applied to another set of sequences or another genome. The human ENCODE regions have a wider range of sequence conservation compared to the *Drosophila*, and the DNase I hypersensitive sites are not biased toward developmental enhancers. These regions have been heavily studied in the past few years so there are plenty of annotations and there are going to be more. Our results show how the performances of some methods change depending on the composition and structure of genomes. This suggests that a method developed for a general purpose, regardless the genome, needs to be tested on multiple genomes to show its general applicability.

Certainly this assessment and analysis are also only based on the available annotations, such as the cell type dependant DNaseI hypersensitive sites [70] we used as potential CRMs, which mark the chromatin regions accessible to all types of proteins but not only limited to transcription factors. There is no direct equivalent CRM database to REDfly in mammals. In addition, the parameter settings of the methods are their defaults, and might not be the optimal settings for some methods to show their peak performance.

### Future directions

The major difficulty of modelling CRMs comes from the fact that the majority of direct and indirect interaction relationships between transcription factors remain unknown. These subtle but critical transcriptional regulatory codes might only be decoded on a smaller scale: such as using expression microarrays or RNA-seq to identify the co-regulated genes then extracting the common patterns from the upstream of these co-regulated genes, or identifying the interaction relationship within a module through a gene regulatory network analysis.

Even with the interaction relationships known, the dynamic information at different conditions are needed to really understand the regulation machinery. The transcriptional logic code is sensitive to conditions. Depending on the context, cis-regulatory elements can be active for function or not, and can perform different roles too: either as transcription factor binding sites, or as facilitated steps for CRM scanning along the sequence or looping and tethering intervening DNA [71].

So far, among all the methods studied in this assessment, only EEL takes DNA structure of a sequence into consideration. Recently, other types of information have been used to assist the CRM prediction, such as the DNA double helix structure profile [72], chromatin structure and histone modification [73], and chromatin immunoprecipitation followed by microarray analysis (ChIP-chip) [74] or chromatin immunoprecipitation followed by high-throughput sequencing (ChIP-seq) [75]. In anticipation of a large-scale analysis, one of the most intriguing projects, ENCODE Pilot Project, is scaled up to a production phase to annotate the entire human genome. This ongoing project will systematically and comprehensively identify transcription factor binding sites, map the histone modifications, and mark the methylation status of CpG-rich regions (http://www.genome.gov/10005107). In addition, the modENCODE project will identify these regulatory elements on the *Drosophila* and worm genomes [76]. During this process, the existing technologies including DNaseI hypersensitivity assays and chromatin immunoprecipitation followed by high-throughput sequencing are applied, whereas more advanced high-throughput computational and experimental methods are in great demand. To answer this request, novel analysis strategies and prediction methods that integrate sequence information and chromatin signatures could be a major step forwards. For instance, Won et al. [77] integrated strong Histone H3 Lys 4 methylations (H3K4me1/2/3) signals together with sequence affinity for transcription factor binding sites into one hidden Markov model to characterize regulatory regions on mouse embryonic stem cells. We believe with the assistance of new technologies, novel analysis strategies, and more complete functional annotations, next generation CRM prediction methods will aim to recreate a dynamic picture of transcription regulation interactions in three-dimensional space. Beyond identifying CRM locations, the future focus will also turn to measuring and predicting spatio-temporal cis-regulatory activity [74], [78], [79].

### Guide for users

For the *Drosophila* genome, based on the results of the REDfly database, which possibly promotes bias toward methods relying on sequence conservation, MorphMS produces the most successful and stable predictions when dealing with the non-exonic regions. The peak phastCons score window method with 500 bp setting can be a good choice too but users may need to double check to confirm the predicted regions are indeed functional as CRMs. Other methods can be used here to provide this information by checking which transcription factors bind there. ClusterBuster is the best choice for single genome, or MorphMS for multiple genomes. However, users need to be aware that the predefined motif library limits the performance of both ClusterBuster and MorphMS. They cannot predict successfully on a region with unknown transcription factor binding sites. Even for the known transcription factor binding sites, there might be a disagreement between the transcription factor binding site profile provided and the genuine transcription factor binding sites on the sequence.

For those regions with unknown transcription factor binding sites, CisPlusFinder appears to offer a solution, by searching for multiple conserved, locally overrepresented sequences as potential binding sites. Therefore there will not be any constraints due to lack of prior knowledge of these binding sites. One condition for CisPlusFinder to locate a potential CRM is the existence of multiple homotypical clusters. This causes CisPlusFinder to miss all CRMs interacting with only one transcription factor, or a single binding site of every transcription factor it contains. Another issue is that a real transcription factor binding site signal might not be abundant in one particular CRM; therefore the perfect local ungapped sequences might not be able to represent all the transcription factor binding sites.

For this reason CisPlusFinder can be used combined with ClusterBuster or MorphMS to discover every CRM candidate. These two different families methods are not only complementary to each other on searching for the unknown transcription factor binding sites, but also on searching for different lengths CRMs: the probabilistic modelling family methods tend to find short CRMs, while CisPlusFinder tend to find long CRMs.

For the mammalian genome, the peak Regulatory Potential score window method is the best way to locate CRM regions. ClusterBuster and MorphMS can be used in addition to identify which transcription factors bind there.

## Materials and Methods

### True positive CRM sequence set

REDfly version 2.0 is a curated collection of known *Drosophila* transcriptional cis-regulatory modules and transcription factor binding sites. It contains all experimentally verified *Drosophila* regulatory elements along with their DNA sequences, their associated genes, and the expression patterns they direct. There are in total 665 CRMs and 941 transcription factor binding sites annotation. The first and the third quartile of the length of these CRMs are 907 bp and 2967 bp, and the median is 1520 bp. Because the boundaries of these CRMs are not certain, each CRM region was extracted including its core sequence plus 200 bp flanking regions on both upstream and downstream. Multiple alignments of 12 *Drosophila* species were extracted for each REDfly CRM region. These raw multiple alignments for comparative analysis were produced by Colin Dewey in Lior Pachter's group at UC Berkeley by their multiple-sequence aligner – MAVID [47], based on the first freeze of all the comparative assemblies of 12 *Drosophila* genomes in December 2005 and January 2006 [80].

To make the dataset compatible with all the selected methods requirements, among the 665 CRM sequences, we chose 244 non-redundant CRMs satisfying the following two requirements:

1. The length of the CRM sequence is greater or equal to 100bp.
2. The CRM sequence has alignments of all 12 *Drosophila* genomes in MAVID.

### True negative sequence sets from Drosophila

The four negative sequence datasets: short introns, exons, medium lengh introns and intergenic regions, were extracted from the *Drosophila Melanogaster* genome sequences, where no regulatory elements are supposed to exist. These negative sequences would differ from CRM sequences in their compositional contents, conservation rates, GC content and other features.

The intron dataset was assembled from introns between 12 bp to 81 bp in length. The exon dataset was assebmled from randomly selected exons. For each short intron or exon sequence, 6 bp was removed from its 5′ end and 3′ end to avoid any consensus splice donor sites (GTA/GAGT for intron and G/A for exon) and any consensus splice acceptor sites (C/TAG for intron and C/AAG for exon) [81]. The sequences of each type of source were then randomly selected and randomly extracted, then were concatenated into 244 sequences with the same lengths as the 244 CRMs.

The medium length intron dataset was assembled from introns between 300 bp and 1 kb in length. For each sequence, 150 bp was removed from its both 5′ and 3′ ends to minimize the risk of contamination with any splice regulatory sequences. The integenic dataset was assembled from those integenic regions between 2 kb and 100 kb in length. For each intergenic sequence, 1kb was removed from both its 5′ end and its 3′ end to avoid any promoter sites and post-transcriptional modification sites.

For those methods based on multiple genomes, pairwise alignment of *Drosophila Melanogaster* on *Drosophila Pseudoobscura* of both positive and negative datasets were extracted from MAVID. The alignments of human and mouse were downloaded from the UCSC genome browser. It is from the December 2007 ENCODE Multi-Species Sequence Analysis (MSA) sequence freeze, which consists of orthologous sequences in mouse to the human ENCODE regions

### The peak score window method

The peak phastCons score window method and the peak Regulatory Potential score window method follow the window size settings and the threshold cut-off settings as described in [27]. For phastCons score, a 100 bp window having an average score over 0.13 is counted as a positive; continuous overlapped positive windows are counted as a regulatory region. Same process is applied to Regulatory Potential score, with the cut-off threshold set to be 0.

### Conservation of transcription factor binding sites, CRMs, and noncoding regions of Drosophila genome and ENCODE regions

For the Drosophila genome, the conservation degrees were checked for the ChIP-on-chip verified transcription factor binding sites of four transcription factors (http://furlonglab.embl.de/data/download): *Mef2* [82], *Twist* [82], *Bagpipe* and *Biniou* [83]; the REDfly CRMs; and the entire *Drosophila Melanogaster* non-coding regions. For the ENCODE regions, the conservation degree were checked for the ENCODE Yale/UC-Davis/Harvard TFBSs by ChIP-seq of eight transcription factors (http://genome.cse.ucsc.edu/cgi-bin/hgTrackUi?db=hg18&g=wgEncodeYaleChIPseq): *c-Fos*, *c-Jun*, *c-Myc*, *GATA-1*, *JunD*, *Max*, *NF-E2* and *ZNF263* [84]; the ENCODE Regulome DNase I hypersensitive sites and the entire ENCODE non-coding regions.

## Supporting Information

Table S1 Prediction scores of the 19 annotated CRMs.
(0.03 MB XLS)

Figure S1 Predictions on ENCODE regions with multiple window size settings. The increase of the window size universally increased the performance of the selected methods.
(0.71 MB TIF)
