# Supplementary figures and images for: Assessing Computational Methods of Cis-Regulatory Module Prediction
Source: PLoS Comput Biol. 2010 Dec 2;6(12):e1001020. doi: 10.1371/journal.pcbi.1001020 (PMC2996316; doi:10.1371/journal.pcbi.1001020)

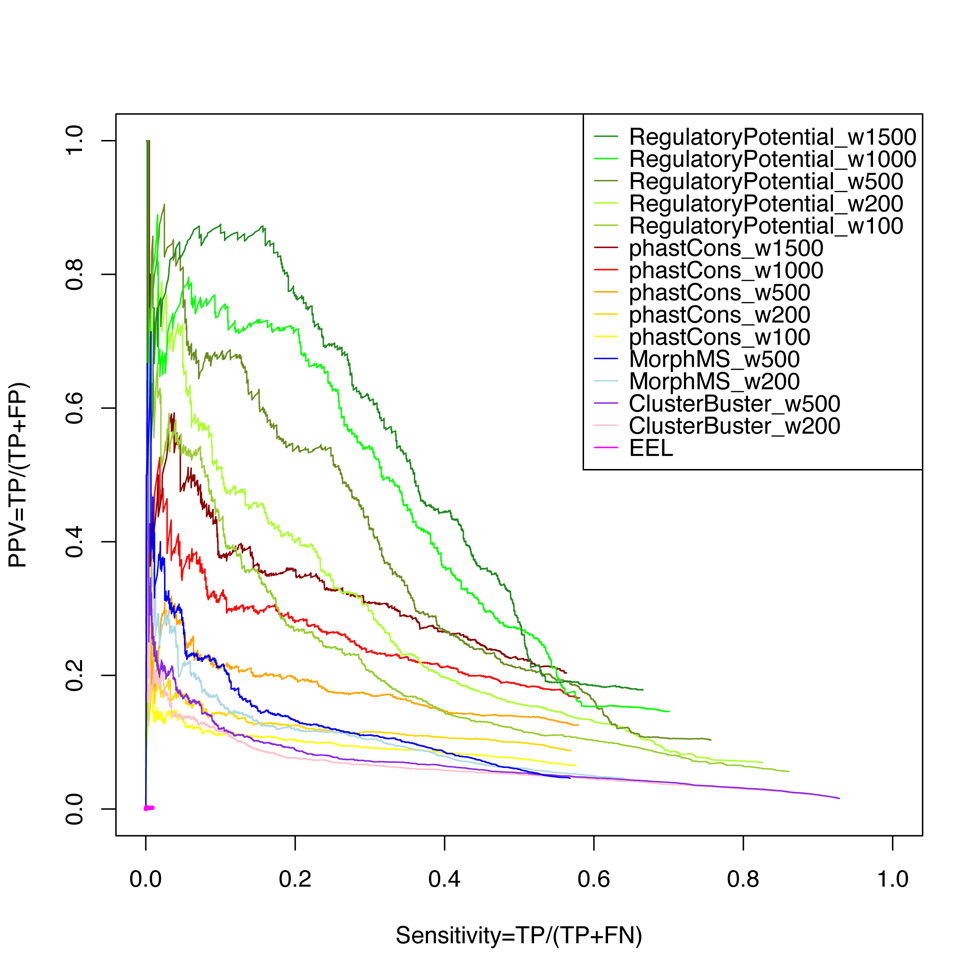

Supplement: Figure S1 — Predictions on ENCODE regions with multiple window size settings. The increase of the window size universally increased the performance of the selected methods. (0.71 MB TIF) [file pcbi.1001020.s002.tif]
